# Restoration of renal TIMP3 levels via genetics and pharmacological approach prevents experimental diabetic nephropathy

**DOI:** 10.1002/ctm2.305

**Published:** 2021-02-04

**Authors:** Viviana Casagrande, Giulia Iuliani, Stefano Menini, Giuseppe Pugliese, Massimo Federici, Rossella Menghini

**Affiliations:** ^1^ Departments of Systems Medicine University of Rome “Tor Vergata” Rome Italy; ^2^ Research Unit of Diabetes and Endocrine Diseases Fondazione IRCCS “Casa Sollievo della Sofferenza” San Giovanni Rotondo Italy; ^3^ Department of Clinical and Molecular Medicine “Sapienza” University Rome Italy; ^4^ Center for Atherosclerosis Department of Medical Sciences Policlinico Tor Vergata University Rome Italy

**Keywords:** chronic kidney disease, diabetes, metalloproteinase, peptide

## Abstract

**Background:**

Diabetic nephropathy (DN), one of the major complications of diabetes, is characterized by albuminuria, glomerulosclerosis, and progressive loss of renal function. Loss of TIMP3, an Extracellular Matrix bound protein affecting both inflammation and fibrosis, is a hallmark of DN in human subjects and mouse models.

**Methods:**

This study was designed to provide evidences that the modulation of the system involving TIMP3 and its target A Disintegrin And Metalloproteinase 17 (ADAM17), may rescue kidney pathology in diabetic mice. Mice with cell‐targeted overexpression of TIMP3 in myeloid cells (MacT3), podocyte‐specific ADAM17 knockout mice (∆PodA17), and DBA/2J mice, were rendered diabetic at 8 weeks of age with a low‐dose streptozotocin protocol. DBA/2J mice were administered new peptides based on the human TIMP3 N‐terminal domain, specifically conjugated with G3C12, a carrier peptide highly selective and efficient for transport to the kidney. Twelve weeks after Streptozotocin injections, 24‐hour albuminuria was determined by ELISA, kidney morphometry was analyzed by periodic acid‐shift staining, and Real Time‐PCR and western blot analysis were performed on mRNA and protein extracted from kidney cortex.

**Results:**

Our results showed that both genetic modifications and peptides treatment positively affect renal function and structure in diabetic mice, as indicated by a significant and consistent decline in albuminuria along with reduction in glomerular lesions, as indicated by reduced mesangial expansion and glomerular hypertrophy, decreased deposition of extracellular matrix in the mesangium, diminished protein expression of the NADPH oxidases 4 (NOX4), and the improvement of podocyte structural markers such as WT1, nephrin, and podocin. Moreover, the positive effects were exerted through a mechanism independent from glycemic control.

**Conclusions:**

In diabetic mice the targeting of TIMP3 system improved kidney structure and function, representing a valid approach to develop new avenues to treat this severe complication of diabetes.

## Graphical abstract

• Loss of TIMP3 contributes to the onset and progression of diabetic nephropathy in human and mouse.

• Strategies aimed to increase TIMP3 activity in the diabetic kidney positively affect renal structure and function.

• Exogenous TIMP3 peptides, specifically targeted to the kidney, may represent a therapeutic approach for the treatment of diabetic nephropathy.

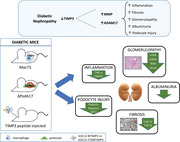

## 1 BACKGROUND

Diabetic nephropathy (DN) is a major complication of type I and II diabetes and the main cause of end stage renal disease (ESRD). Nephropathy occurs in 20‐40% of all diabetics and as many as 44% of patients undergoing dialysis are diabetic.^1^ The clinical and pathological hallmarks of DN are a gradual decline in glomerular filtration rate (GFR) and progressive albuminuria. Morphologically, DN is characterized by glomerulosclerosis, a result of increasing accumulation of extracellular matrix and podocyte dysfunction.^2^ Despite current therapies available for controlling hyperglycemia and hypertension, additional treatment strategies are required to reduce the incidence of diabetes‐related ESRD. Tissue Inhibitor of Metalloproteinase 3 (TIMP3) is an ExtraCellular Matrix (ECM)‐bound protein that is broadly expressed in humans and mice and is involved in a wide range of physiological processes, such as cell growth and migration, angiogenesis and apoptosis. TIMP3 is the most highly expressed TIMP in the kidney and has a broad protease inhibition profile, and we and others have already demonstrated that loss of TIMP3 contributes to the onset and progression of DN in mouse models of diabetes and in human patients.^3^, ^4^, ^5^, ^6^, ^7^ TIMP3 is the only known physiological inhibitor of A Disintegrin And Metalloproteinase (ADAM) 17, a metalloprotease responsible for shedding of several ligands involved in the pathogenesis of chronic kidney disease and glomerulonephritis.^8^, ^9^ Several stimuli can increase ADAM17 activity in a tissue or cell‐specific manner.^10^, ^11^ ADAM17 expression and activity are increased in the kidney cortex of OVE26 mice with type 1 diabetes and in renal cells exposed to high glucose concentrations,^4^ and high ADAM17 mRNA staining was observed in the glomerular parietal epithelium and podocytes from several renal diseases, including DN.^12^ Elevated plasma concentrations of tumor necrosis factor receptor 1 (TNFR1) and tumor necrosis factor receptor 2 (TNFR2), two ADAM17 substrates, have been recently found to predict stage 3 chronic kidney disease (CKD) and ESRD in patients with type 1 and type 2 diabetes, respectively.^13^, ^14^ Among TIMP3 targets, matrix metalloproteinases (MMPs) affect the breakdown and turnover of ECM and altered MMPs expression and activity contribute to DN.^15^ In particular, MMP9 has been shown to be involved in dedifferentiation of podocytes and renal tubular cells in diabetic mice, and its expression is increased in glomeruli from diabetic patients.^16^ This evidence suggests the relevance of a tightly regulated balance between TIMP3 and its targets in the control of kidney fibrosis during DN.

Many proteins and peptides take on an increasingly important role as therapeutics in areas including diabetes diseases mainly due to their potential to bind to targets in a selective manner, thus reducing undesired off‐target effects.^17^ Drug‐targeting delivery to the kidney is an attractive method to increase the pharmaceutical compounds efficacy,^18^ however it represents a promising but underdeveloped area. Recently, galectin‐3 targeted G3C12 peptide and ε‐polylysine derived, synthetic (KKEEE)3K peptide have been identified as novel highly kidney‐specific carriers with exceptional kidney selectivity, even with conjugated drugs, rapid kidney accumulation, and no toxic profile.^19^, ^20^ Here, we provide evidence that human TIMP3 overexpression in myeloid lineage, conditional deletion of ADAM17 in podocytes, and intervention with innovative kidney‐targeted TIMP3 derived peptides, result in protection from renal damage in experimental models of DN. Together, these findings suggest that the modulation of the system involving TIMP3 and its targets, may rescue kidney defects in diabetic mice, with implications for future therapeutic interventions in DN.

## 2 MATERIALS AND METHODS

### 2.1 Animal models

Animal procedures were approved by local and national committees in charge (Tor Vergata University Institutional Animal Care and Use Committee and Ministry of Health, license no. 378/2016‐PR and 36/2019‐PR) and conducted in accordance with accepted standard of humane animal care.

MacT3 transgenic mice, overexpressing TIMP3 under the control of the monocyte/macrophage lineage‐specific promoter CD68, were previously described,^21^, ^22^ and littermate C57BL/6J mice (wt) were used as controls.

Homozygous Adam17flox/flox (referred to as Ct) were mated with B6.Cg‐Tg(NPHS2‐Cre)295Lbh/J purchased from The Jackson Laboratory to generate Nphs2‐Cre/ Adam17flox/flox mice (referred to as ΔPodA17). These mice have been backcrossed for >6 generations and are congenic on a C57BL/6 background. Mouse DNA was isolated from tail, and genotype was analyzed by polymerase chain reaction (PCR).

Six‐week‐old male DBA/2J mice were purchased from Charles Laboratories and were maintained in a controlled environment for 2 weeks in order to acclimate them to local conditions.

Male C57Bl/6J, MacT3, Ct, ΔPodA17, and DBA/2J mice were maintained in our animal facility (12 hours light/dark cycle; 22°C ± 1 °C, 50% ± 5% humidity) and fed ad libitum with standard laboratory chow (Mucedola s.r.l.). At the end of each experiment, mice were sacrificed by cervical dislocation; blood and tissues were collected for biochemical and morphological analyses.

### 2.2 Type 1 diabetes model and experimental protocols

Diabetes was induced in 8‐week‐old mice by a multiple low‐dose streptozotocin (STZ) (Sigma) injection. STZ was given intraperitoneally (ip) at 50 mg/kg per day (freshly made in Na‐Citrate buffer, pH 4.5) for 5 consecutive days after a 4 hours fast. Control animals were treated with Na‐citrate buffer alone. Diabetes was defined as fed blood glucose level ≥250 mg/dL using a glucometer (OneTouch Ultra, LifeScan).

C57Bl/6J, MacT3, Ct, and ΔPodA17 mice were sacrificed 13 weeks after the final injection of STZ.

At 5 weeks after STZ or Na‐Citrate buffer administration, DBA/2J mice were randomly divided into the following four treatment groups: (1) vehicle buffer (PBS), (2) G3C12 free peptide, (3) G3C12‐NTIMP3 peptide, and (4) G3C12‐T2GNTIMP3 peptide. Peptides were dissolved in sterile PBS and administered intravenously (iv) by retro‐orbital injection (2 mg/kg body weight) in mice two times per week for 8 weeks.

At the end of each experiment, individual mice were placed in metabolic cages for 24 hours to obtain urine collections.

### 2.3 Peptides synthesis

All peptides (50 mg, purity > 95%) were generated by ProteoGenix SAS by chemical synthesis from the C‐terminal extremity to the N‐terminal extremity. The peptides were purified using preparative high performance liquid chromatography (HPLC) (Kromasil 100–5). Quality of peptides was analyzed by HPLC (LC3000) and Mass Spectrometry (Shimadzu LCMS‐2020). NTIMP3 and T2GNTIMP3 consisted in the 120‐amino acid peptide corresponding to loop 1 of human TIMP3 protein, in a native form or in a form with a T2G point mutation. NTIMP3 and T2GNTIMP3 were conjugated at the N‐terminal site with G3C12 carrier peptide (ANTPCGPYTHDCPVKR) to obtain G3C12‐NTIMP3 and G3C12‐T2GNTIMP3. T2GNTIMP3 was conjugated at the N‐terminal site with (KKEEE)3K carrier peptide to obtain (KKEEE)3K‐T2GNTIMP3.

A conjugation of G3C12 carrier peptide at the N‐terminal site of N‐terminal TIMP3 protein in a native form containing the inhibitor activity sites for MMPs and ADAM17 (NTIMP3), or in a form with a point mutation (T2G) described as able to uphold the inhibitory activity of ADAM17 (T2GNTIMP3).^23^

### 2.4 TNF‐α, TNFR1, and albumin quantification

Measurements of tumor necrosis factor alpha (TNF‐α) and TNFR1 concentration in mice sera and urines were obtained using specific ELISA kits (R&D Systems), according to the manufacturer's protocol.

Urine albumin was determined using a mouse albumin ELISA kit (Abcam) according to the manufacturers’ instructions.

### 2.5 In vivo pharmacokinetics studies

To determine the appropriate dose and injection site for the peptide, iv, subcutaneously (sc), and ip injections with 0.5, 1, or 2 mg/kg body weight of G3C12‐NTIMP3 peptide or PBS (n = 5 DBA/2J mice per group) were performed. Mice were sacrificed 24 hours post‐injection, blood and kidneys were collected.

For the study of tissue distribution, serum, kidney (cortex and medulla), liver, pancreas, muscle, and aorta were collected at different time points (0, 10 minutes, 30 minutes, 1, 3, 6, 24, 48, and 72 hours) after 2 mg/kg body weight of G3C12‐NTIMP3 peptide (n = 3) iv injection.

### 2.6 Isolation of mouse glomeruli

Mouse glomeruli were isolated by Percoll‐based density gradient. Kidneys were harvested, and the medulla was removed. The renal cortexes were minced into small pieces by cutting with scissors in 0.5 mL ice‐cold HBSS (Life Technologies) and subjected to digestion in 2 mL collagenase type IV (Sigma, 1 mg/mL dissolved in HBSS) at 37°C for 30 minutes with gentle shaking.

The digested tissue was transferred onto a prewetted 100 μm mesh stainless steel cell strainer and centrifuged at 600 × g for 5 minutes at 4°C. The supernatant was discarded, and the tissue rapidly resuspended in 5 mL of ice‐cold HBSS for three times.

Density‐gradient centrifugation of the pellet was then performed by resuspension in 10 mL of 45% (vol/vol) sterile Percoll solution in 50 mL centrifugation tubes and centrifugation at 20000 × g for 45 minutes at 4°C (without braking). After centrifugation, the tubule fractions with heavy enrichment in glomeruli were collected from the top layer of the Percoll solution. The tubule fraction was washed once in 5 mL ice‐cold HBSS at 600 × g for 5 minutes at 4°C and resuspended for further experiments.

### 2.7 In vitro MMP2/9 inhibitor screening and ADAM17 activity assay kits

The MMP2/9 activities were evaluated using MMP2/MMP9 colorimetric Inhibitor Screening Assay kits (Abcam) under the manufacturer's instruction. In brief, 0.9 U of human recombinant MMP2 or MMP9 was mixed with 1 μM G3C12, G3C12‐NTIMP3, G3C12‐T2GNTIMP3 peptides, or 1.3 μM NNGH inhibitor as negative control. The mixture was incubated at 37°C for 60 minutes then substrate was added. The plate was read at 412 nm by infinite F50 microplate reader (TECAN). Continuous recording was made at 1 minute interval for 20 minutes. The experiment was performed in triplicate.

ADAM17 activity was determined using the SensoLyte 520 TACE Activity Assay Kit (AnaSpec). 1 μM human recombinant ADAM17 (R&D Systems) was incubated with the QXL520/ 5‐FAM FRET substrate, derived from a sequence surrounding the cleavage site of ADAM17. For inhibitor studies, 1μM TAPI‐0 or 1 μM G3C12, G3C12‐NTIMP3 and G3C12‐T2GNTIMP3 peptides were used. Active ADAM17 cleaves FRET substrate into two separate fragments resulting in an increase of 5‐FAM fluorescence which can be monitored at excitation/emission = 490 nm/520 nm measured in a fluorescence microplate reader (Beckman Coulter DTX 800) continuously every 5 minutes for 60 minutes (10).

### 2.8 TIMP3 ELISA assay

The TIMP3 expression was determined by human TIMP3 ELISA kit (Biosensis) under the manufacturer's instruction. Undiluted serum or 40 μg cortices homogenized in ice cold PBS were added to the microwell plate pre‐coated with anti‐human TIMP3 antibody and incubated at 4°C overnight on a shaker. Then wells were washed five times allowing the wash buffer to remain in the wells for 2 minutes, and biotinylated antibody working solution was added into each well. The plate was incubated at room temperature on a plate shaker for 3 hours. Then wells were washed three times, and the Avidin‐Biotin‐Peroxidase Complex was added into each well, and the plate was at room temperature on a shaker for 1 hour. Then plate was washed three times, and TMB substrate solution was added and incubated 30 minutes at room temperature in dark. At the end of incubation, 100 μL stop solution was added to each well, and the absorbance at 450 nm was read using infinite F50 microplate reader (TECAN). All experiments were conducted in triplicate.

### 2.9 Renal tissue histology, immunohistochemistry, immunofluorescence, and morphometric analysis

In the right kidney was dissected, weighed, cut sagittally into two halves, and one half was fixed in phosphate‐buffered paraformaldehyde solution (4% [vol./vol.]). After 16 hours fixation, a portion of the kidney tissue was embedded in optimized tissue compound (Tissue Tek) and stored at −80°C until sectioning, and the remaining portion was embedded in paraffin for histological and morphometric analysis. For the analysis of renal structure, serial 4 μm kidney sections were stained with Periodic Acid–Schiff (PAS), as previously reported.^24^ The areas of at least 60 glomerular tuft profiles per sample were measured by means of the interactive image analysis system Image Pro Premier 9.2 (Immagini&Computer), and the mean Glomerular Area (mGA) was estimated from it. The percentage of the glomerular positive area for PAS staining (fractional Mesangial Area [fMA]) was estimated at a fixed color threshold by first defining the glomerulus as a region of interest, which was set by identifying three to five separate pixels in areas of PAS positive staining. Finally, the mean Mesangial Area (mMA) was calculated by the formula: (fMA × mGA)/100.

CD68 positive cells were analyzed by immunohistochemistry (IHC) using a rat monoclonal to CD68 (Abcam, ab53444), followed by a secondary byotinilated goat anti‐rat (Abcam, ab6844). For CD68+ cell infiltration, 20 random fields of the renal cortex were examined at a final magnification of 400X, and the results were expressed as the mean number of CD68 positive cells per field. Protein content and distribution of Type IV Collagen (Coll‐IV) and NADPH Oxidase 4 (Nox4) were assessed by IHC using a rabbit polyclonal to Coll‐IV (Abcam, ab6586)and a rabbit monoclonal to Nox4 (Abcam, ab133303),^24^, ^25^ respectively, followed by the Ultra Teck Anti‐polyvalent Biotinylated Anti‐IgG antibody (ScyTek Laboratories). To assess Nox4 antibody specificity, normal liver tissue, which is known to express very low levels of Nox4, stained with rabbit monoclonal to Nox4 (Abcam, ab133303) and diabetic kidney, in which the primary specific antibody was replaced with the recombinant Rabbit IgG, monoclonal (EPR25A) ‐ isotype control (ab172730) were used as negative controls. As for PAS positivity, the percentage of the glomerular area with positive staining (brown color) was calculated in at least 60 glomeruli of each specimen. Caspase‐3 was analyzed by IHC using a rabbit polyclonal IgG antibody (Promega, Anti‐ACTIVE Caspase‐3), followed by a secondary biotinylated goat anti‐rabbit (Dako, E0432). Positive cells for active‐caspase‐3 were counted and expressed as percent of total glomerular cells. Glomerular staining for ADAM17 and the podocyte marker Wilms Tumor 1 (WT1) was evaluated by dual label immunofluorescence using a rabbit polyclonal to ADAM17 (Abcam, ab2051), and a goat polyclonal to WT1 (Santa Cruz Biotechnology, sc‐15421), followed by the secondary fluorescent‐labeled antibodies DyLight488 Anti‐Rabbit IgG (Vector Labs, DI‐1488), and Alexa Fluor 594 Chicken Anti‐Goat IgG (Invitrogen, A21468), respectively. Section analysis was performed on a fluorescence microscope (Zeiss AXIO A1), equipped with an Axiocam 503 color camera (Carl Zeiss Italy).

### 2.10 Immunoblotting

Kidney cortex slices or glomeruli were homogenized in ice‐cold buffer containing 20 mM Tris (pH 7.6), 137 mM NaCl, 1 mM MgCl2, 1 mM CaCl2, 1% Triton X‐100, 10% Glycerol, 2 mM EDTA plus protease inhibitors, for 20 minutes at 4°C, and the remaining debris was cleared by subsequent 20 minutes centrifugation at 12500 rpm, 4°C. The protein concentrations were evaluated by Bradford (BioRad); 40 μg of total protein per lane was diluted in standard SDS sample buffer and subjected to electrophoresis on SDS‐polyacrylamide gels. The following antibodies were used: podocin (sc‐21009), nephrin (sc‐28192), Wilms' tumor 1 (WT1) (sc‐192), transforming growth factor beta (TGF‐β) (sc‐7892), actin (sc‐376421) (Santa Cruz Biotechnologies), Coll‐IV (Thermo Fisher Scientific, PA5‐104508), and TIMP3 (Abcam, ab39184). Quantity One/ImageJ sofware (BioRad) was used for densitometric scanning.

### 2.11 RNA isolation and gene expression analysis

Total RNA was isolated from cortex using TRIzol Reagent (Invitrogen). Quantity of RNA was checked by Nanodrop (Thermo Fisher Scientific), and 2 mg of total RNA were reverse transcribed into cDNA using the High Capacity cDNA Archive Kit (Applied Biosystems). A quantitative real‐time PCR was performed using an ABI PRISM 7700 System and TaqMan reagents (Applied Biosystems). Each reaction was performed in triplicate using standard reaction conditions: 1 cycle at 50°C for 2 minutes, 1 cycle at 95°C for 10 minutes, and 40 cycles each at 95°C for 15 seconds and 60°C for 1 minute. The cycle threshold value was normalized in mouse by b‐actin.^26^ Primer codes: TIMP3: Mm00441826_m1; F4/80: Mm00802529_m1; MCP1: Mm00441242_m1; TNF‐α: Mm00443258_m1; Il6: Mm00446190_m1; COL1A2: Mm00483888_m1 (Applied Biosystems).

### 2.12 Metabolomics

Metabolomics profiling was performed using established protocols (Metabolon).^26^ Following log transformation and imputation with minimum observed values for each compound, Welch's two‐sample *t*‐test was used to identify biochemicals that differed significantly between experimental groups.

### 2.13 Statistical analysis

Results of the experimental studies are expressed as mean ± standard error of the mean (SEM). Statistical analyses were performed using GraphPad Prism (v.5.02). Groups were compared using a two‐tailed unpaired Student's *t* test or one‐way ANOVA with Bonferroni Multiple Comparison Test or Dunnett's Multiple Comparison Test as indicated. Values of p < 0.05 were considered to be statistically significant.

## 3 RESULTS

### 3.1 TIMP3 overexpression in macrophages ameliorates diabetes induced kidney damage

**FIGURE 1:**
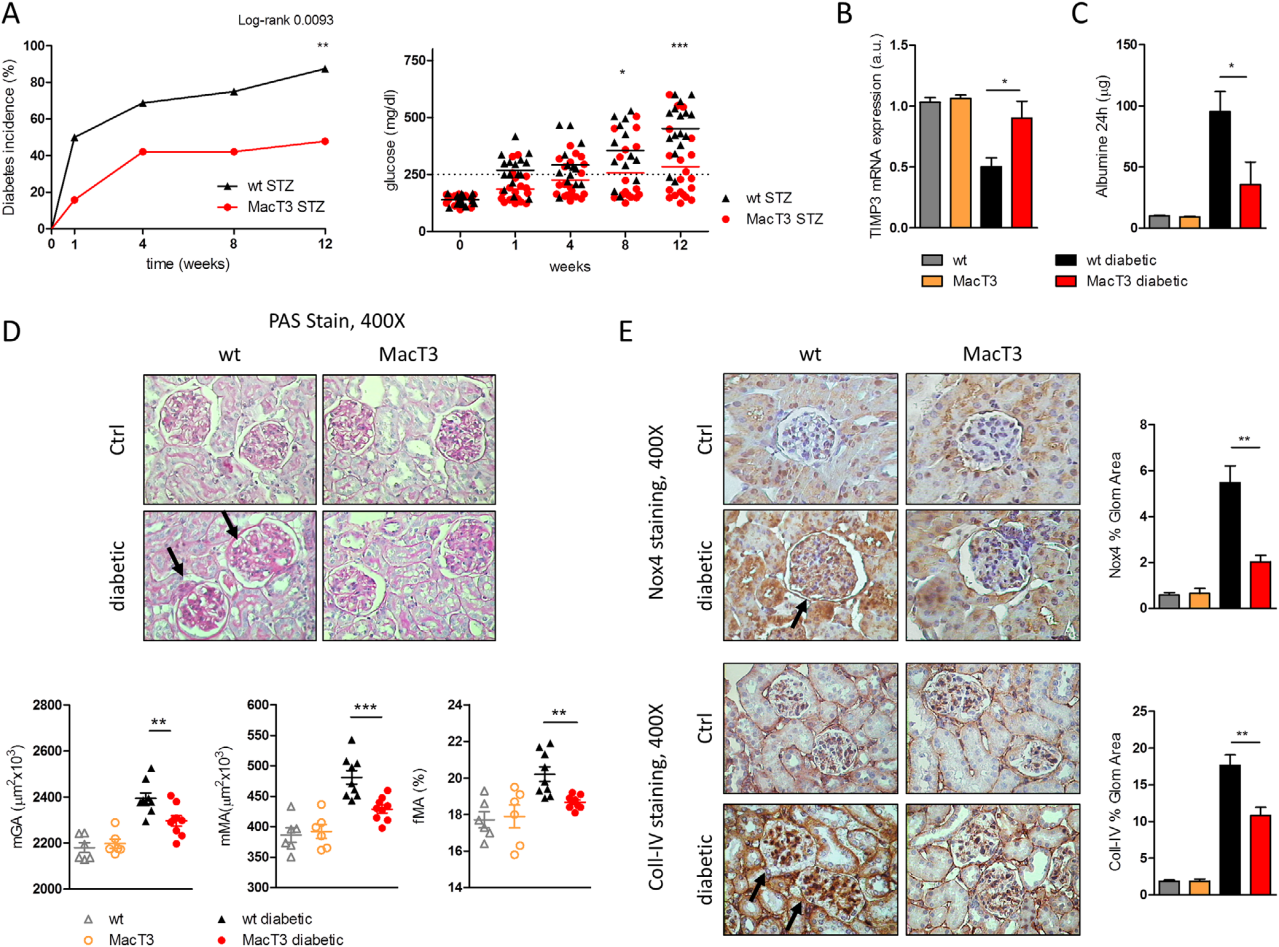
TIMP3 overexpression in macrophages ameliorates STZ‐induced kidney damage. (A) Diabetes incidence in wt and MacT3 mice after STZ treatment (Kaplan‐Meier curves analyzed by Mantel‐Cox log‐rank test). Mice were considered diabetic when the blood glucose levels equaled or exceeded 250 mg/dL on two consecutive tests. Twelve weeks after STZ administration diabetic wt and MacT3 mice and age‐matched non‐diabetic littermates (Ctrl) were analyzed for (B) gene expression of TIMP3 (mRNA expression was determined by real‐time PCR and normalized to β‐actin mRNA) (n  =  6 per group). (C) Twenty‐four hours urinary albumin excretion (n = 6 per group), (D) PAS‐staining in kidney and quantification of mGA, fMA and mMA (arrows show glomerular hypertrophy and mesangial expansion) and (E) immunohistochemical detection of Nox4 and Coll‐IV in kidney (non‐diabetic n = 6, diabetic n = 9 per group, original magnification, X400) (arrows indicate increased glomerular staining for Nox4 and Coll‐IV). (*p  <  0.05, **p ≤ 0.01, ***p ≤ 0.001; Student's *t* test comparing diabetic mice, data are means ± SEM). Abbreviation: a.u., arbitrary unit

**FIGURE 2:**
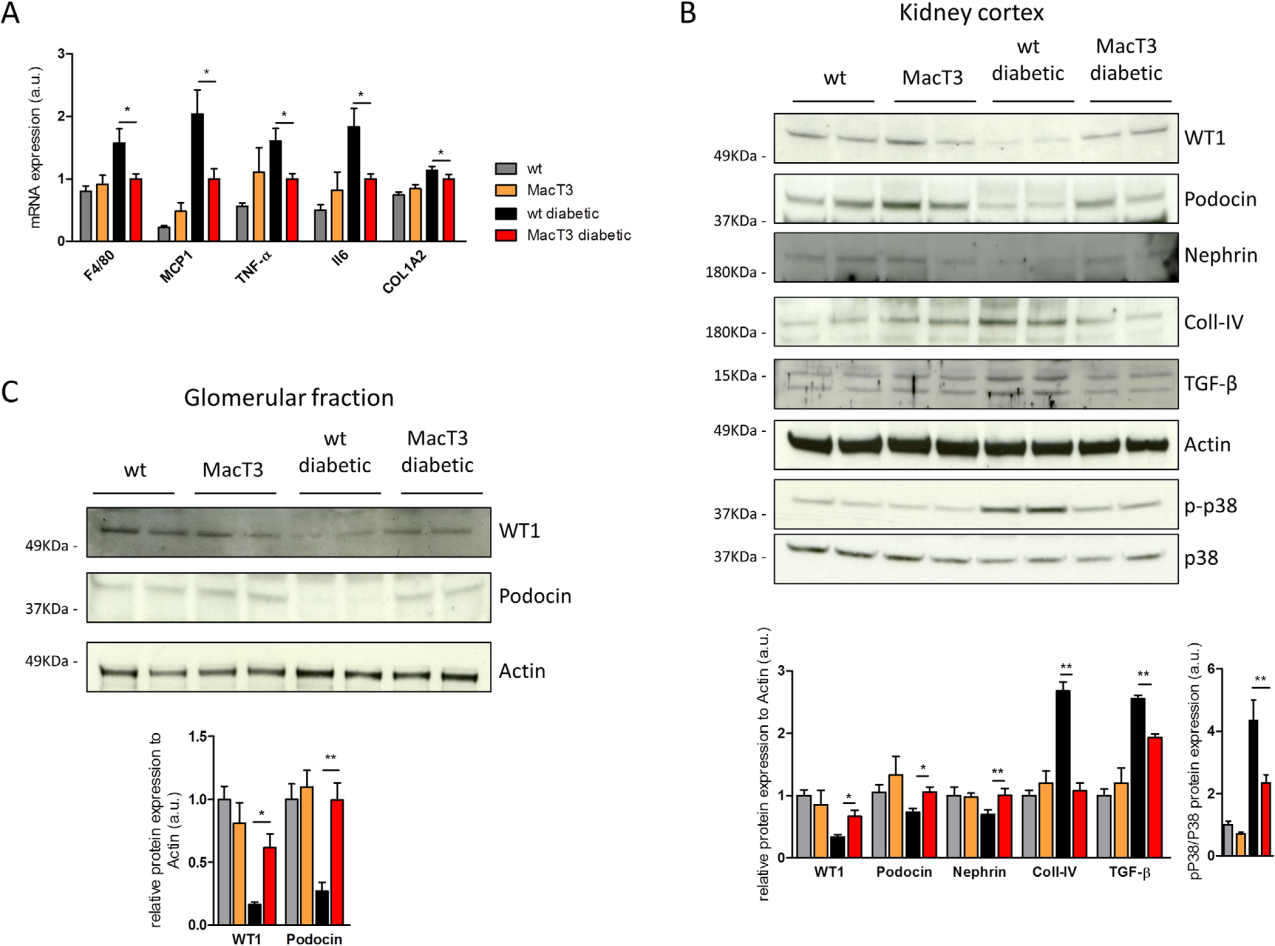
TIMP3 overexpression in macrophages reduces STZ‐induced inflammation and fibrosis in kidney. Kidney cortex analysis of (A) gene expression of F4/80, MCP1, TNF‐α, IL6, and COL1A2 and (B) protein levels of WT1, Podocin, Nephrin, Coll‐IV, TGF‐β, Actin, phospho‐p38 (p‐p38), p38 in wt, and MacT3 non‐diabetic and diabetic mice (n  =  6 per group). (C) Protein levels of WT1, Podocin, and Actin in kidney glomerular fraction of wt and MacT3 non‐diabetic and diabetic mice (n = 5 per group). A representative image of two mice per group is shown. Relative protein levels as determined by densitometry (*p  <  0.05, **p ≤ 0.01; Student's t test comparing diabetic mice, data are means ± SEM). Abbreviation: a.u., arbitrary unit.

First, we analyzed a previously generated mouse model with cell‐targeted overexpression of TIMP3 under CD68 promoter (MacT3).^21^, ^22^ Here, 8‐week‐old male wild type (wt) and MacT3 mice received repeated injection of low dose pancreatic islet cell toxin STZ to induce diabetes.^27^ Blood glucose level was monitored every week. The results showed that 12 weeks after STZ administration, 87% (14/16) of wt mice exhibited hyperglycemia compared to 45% (9/20) of MacT3 mice, indicating that TIMP3 overexpression in cells of the myeloid lineage significantly reduced the susceptibility to type 1 diabetes (p < 0.01) (Figure 1A). For further experiments, only diabetic mice have been considered. Obtained results indicated that after 12 weeks of hyperglycemia, mice showed the same rate of diabetes‐induced CD68 positive cells infiltration in renal cortex (Figure S1), diabetic MacT3 mice showed increased TIMP3 mRNA expression (p < 0.05) (Figure 1B) and developed significantly less albuminuria compared with the diabetic wt mice (p < 0.05) (Figure 1C). Next, histological examination of kidneys was performed by PAS staining. Morphometric analysis showed a significantly reduction in renal lesions in diabetic MacT3 mice, as assessed by lower increase in mGA (p < 0.01), mMA (p < 0.001), and fMA (p < 0.01) (Figure 1D). Reduction of Coll‐IV and Nox4 expression, as shown by immunostaining analysis, suggested a protection from extracellular matrix accumulation and glomerular damage in diabetic MacT3 mice (p < 0.01) (Figure 1E). Both, Nox4 evaluation in normal liver tissue, which is known to express very low levels of Nox4, and the replacement of the primary specific antibody with rabbit nonimmune IgG in diabetic kidney showed no significant staining (Figures S2A‐S2C). The mRNA expression of inflammatory (F4/80), Monocyte Chemoattractant Protein‐1 (MCP‐1), TNF‐α and Interleukin 6 (IL6), and fibrotic Collagen type I alpha 2 (COL1A2) markers was reduced in renal cortex of diabetic MacT3 mice compared to WT diabetic mice (p < 0.05) (Figure 2A). Podocytes markers (WT1, Podocin, and Nephrin) resulted significantly improved, and fibrosis markers (Coll‐IV and TGF‐β) as well as phospho‐p38 MAPK were reduced in renal cortex from MacT3 diabetic mice compared with diabetic wt mice (Figure 2B). Moreover, glomerular fraction analysis confirmed the increase of WT1 and Podocin expression in MacT3 mice (Figure 2C), suggesting that overexpression of TIMP3 by the myeloid lineage may have a protective effect against DN, acting by reducing inflammation and consequent fibrosis.

### 3.2 Podocyte deletion of ADAM17 attenuates diabetic kidney injury

**FIGURE 3:**
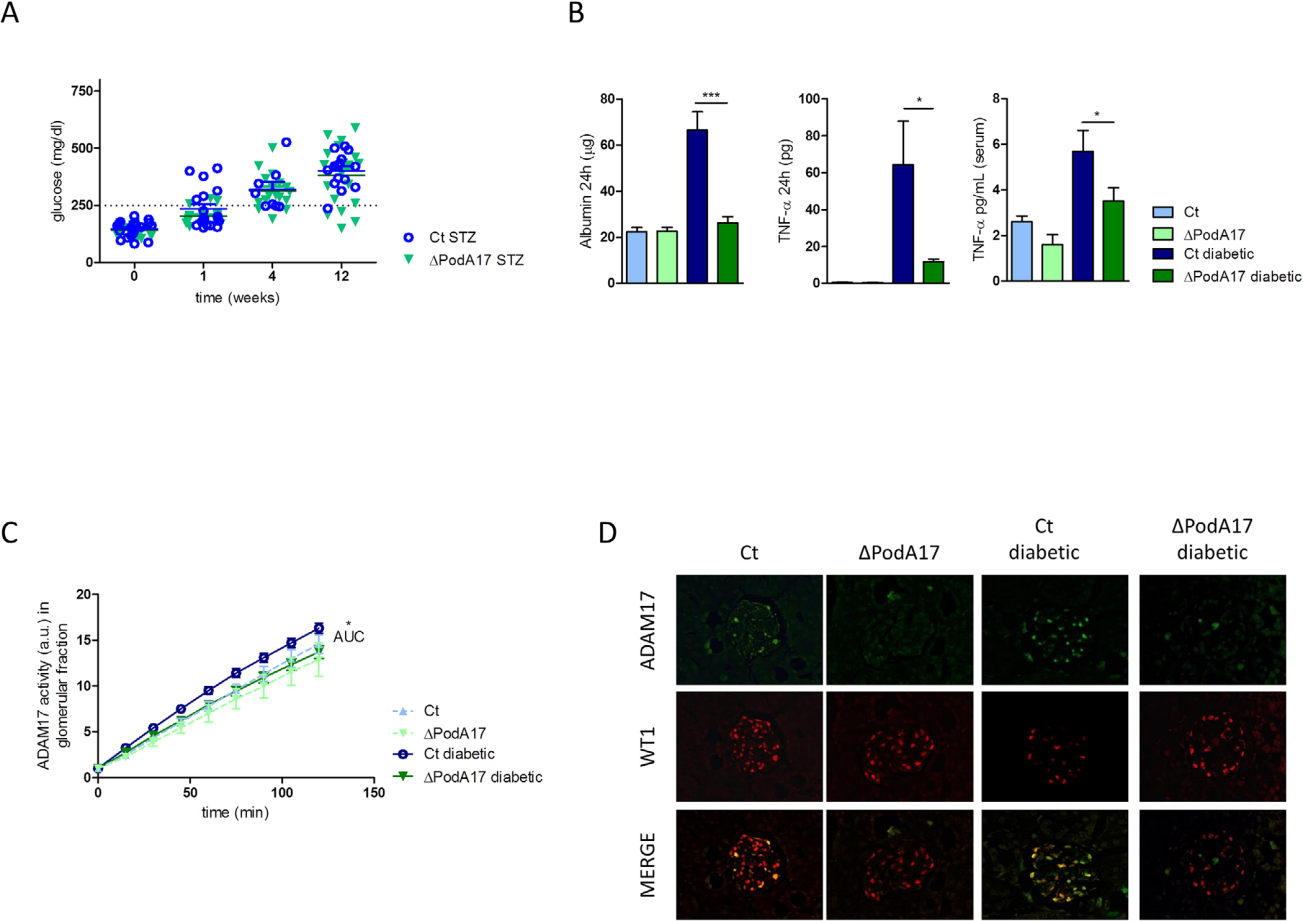
Analysis of diabetic ΔPodA17 mice model. (A) Blood glucose levels at different time points after STZ treatment. Twelve weeks after STZ administration, Ct and ΔPodA17 non‐diabetic and diabetic mice were analyzed for (B) 24‐hour urinary albumin excretion, 24‐hour urinary TNF‐α excretion, and serum TNF‐α level (n = 10 per group). (C) Glomerular ADAM17 activity in Ct and ΔPodA17 non‐diabetic and diabetic mice (n = 6 per group). (D) Immunofluorescence double staining showing expression of ADAM17 and WT1 in Ct and ΔPodA17 mice, and their colocalization (merge). Note the increased staining for ADAM17 in the glomerulus of a Ct diabetic mice and its complete absence in podocytes of ΔPodA17 mice, both diabetic and non‐diabetic. Original magnification, X400 (*p  <  0.05, ***p ≤ 0.001; Student's *t* test comparing diabetic mice, data are means ± SEM)

To address whether specific inhibition of ADAM17 in podocytes contributes to kidney protection during diabetes, we generated mice with a conditionally ablated Adam17 gene in podocytes by crossing mice carrying the floxed ADAM17 allele (Adam17^flox/flox^) with mice expressing the Cre expression cassette under the control of the Nphs2 promoter.^28^ The resulting Nphs2‐Cre/ Adam17^flox/+^ heterozygotes were crossed to produce the desired genotype, Nphs2‐Cre/ Adam17^flox/flox^, here identified as ∆PodA17. These mice were born at the expected Mendelian frequency, indicating that podocyte ADAM17 deletion does not lead to embryonic lethality. Both ∆PodA17 and Adam17^flox/flox^ as a control (Ct) mice were made diabetic by the low dose STZ injections. The two groups of mice developed comparable levels of hyperglycemia within 4 weeks, which persisted through the course of the study (Figure 3A). After 12 weeks from STZ treatment, the diabetic ∆PodA17 mice showed a significant reduction of 24‐hour urinary albumin (p < 0.001) and both urinary and serum levels of TNF‐α than diabetic Ct mice (p < 0.05) (Figure 3B).

**FIGURE 4:**
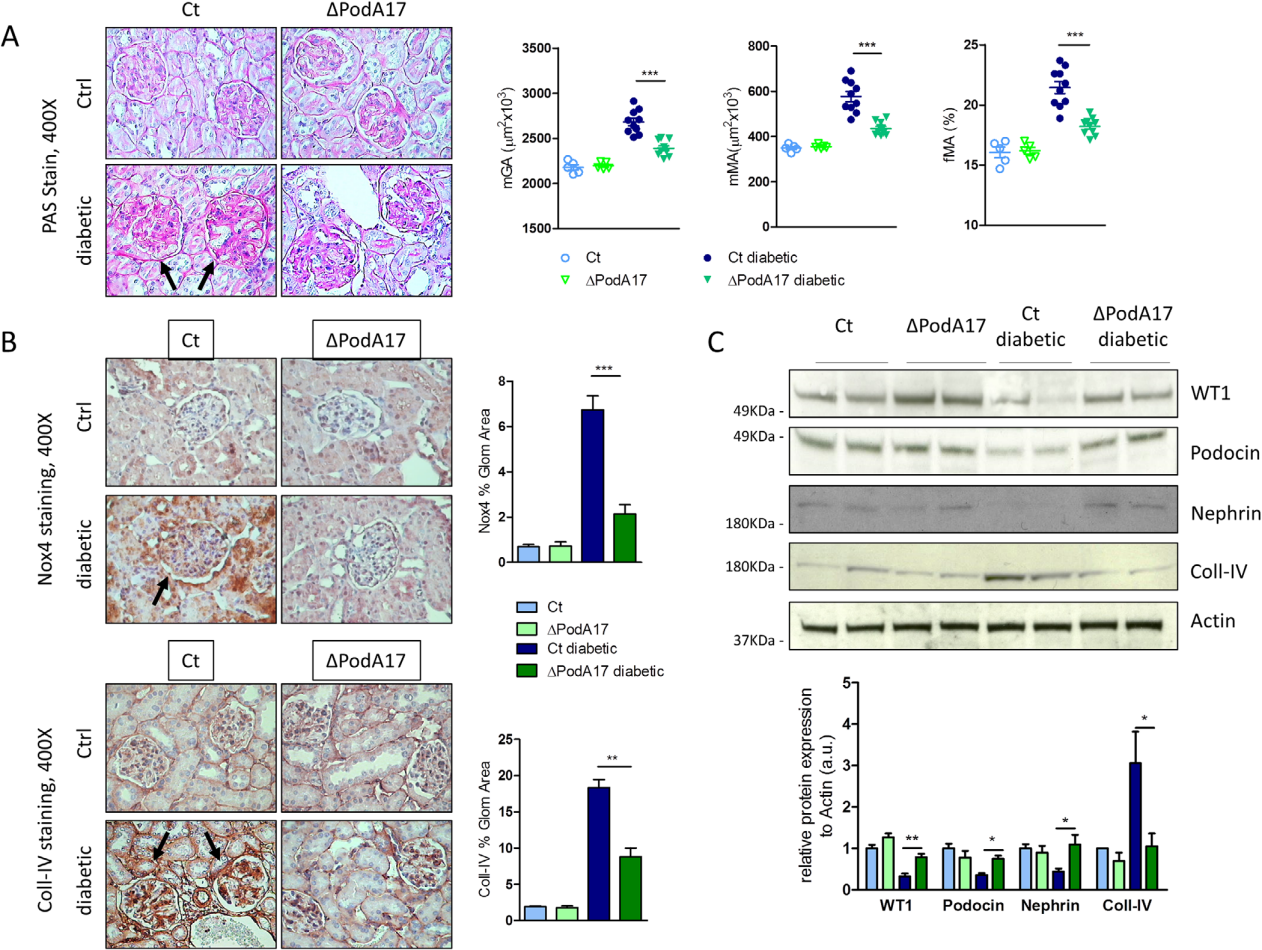
Podocyte deletion of ADAM17 attenuates STZ‐induced injury in kidney. Twelve weeks after STZ administration diabetic Ct and ΔPodA17 mice and age‐matched non‐diabetic littermates (Ctrl) were analyzed for (A) PAS‐staining in kidney and quantification of mGA, fMA, and mMA (arrows indicate glomerular hypertrophy and mesangial expansion) and (B) immunohistochemical staining of Nox4 and Coll‐IV in kidney (non‐diabetic n = 5, diabetic n = 10 per group, original magnification, X400) (arrows indicate increased glomerular staining for Nox4 and Coll‐IV). (C) Protein expression of WT1, Podocin, Nephrin, Coll‐IV, and Actin in kidney cortex from Ct and ΔPodA17 non‐diabetic and diabetic mice (n = 6 per group). A representative image of two mice per group is shown. Relative protein levels as determined by densitometry (*p  <  0.05, **p ≤ 0.01, ***p ≤ 0.0001; Student's *t* test comparing diabetic mice, data are means ± SEM). Abbreviation: a.u., arbitrary unit

Podocyte‐conditional ablation resulted in a remarkable reduction of glomerular ADAM17 activity compared to Ct mice (p < 0.05) (Figure 3C). Immunofluorescence staining revealed that in Ct mice ADAM17 expression was increased in the glomeruli of diabetic compared to normoglycemic mice, particularly in podocytes, whereas in ∆PodA17 mice, complete loss of ADAM17 expression was observed in podocytes of both diabetic and non‐diabetic mice (Figure 3D). This was associated with reduced diabetes‐induced glomerular apoptosis, as indicated by reduced Active caspase 3 levels (Figure S3), and with the decrease in glomerular and mesangial expansion in diabetic ∆PodA17 mice compared with diabetic Ct mice, as determined by PAS staining (p < 0.001) (Figure 4A). ADAM17 ablation in podocytes prevented the increase in extracellular matrix accumulation and glomerular damage, as determined by Coll‐IV (p < 0.01) and Nox4 staining (p < 0.001) (Figure 4B). Consistently, western blot analysis showed that WT1, Podocin, and Nephrin expressions were increased whereas Coll‐IV was reduced in kidney cortex from diabetic ∆PodA17 compared with diabetic Ct mice (Figure 4C). These results indicate that targeting of the ADAM17 pathway may represent a therapeutic target for DN.

### 3.3 G3C12‐NTIMP3 and G3C12‐T2GNTIMP3 characterization in vitro and in vivo

**FIGURE 5:**
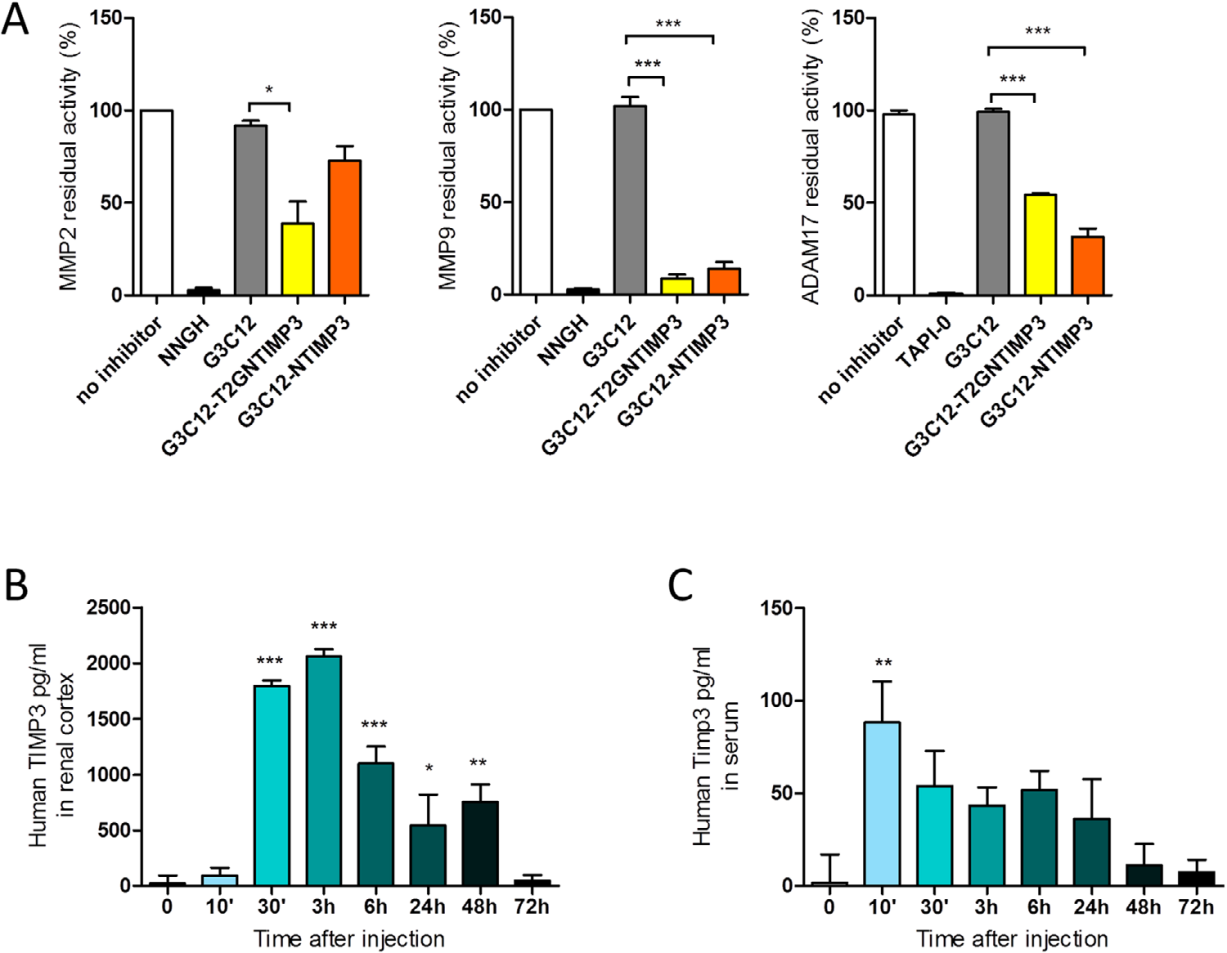
G3C12‐NTIMP3 and G3C12‐T2GNTIMP3 characterization. (A) Inhibition of human MMP2, MMP9, and ADAM17 enzyme activity by G3C12, G3C12‐NTIMP3, and G3C12‐T2GNTIMP3 in vitro. Enzymes were incubated or not with the various peptides (1μM) or positive control (1.3 μM NNGH inhibitor or 1 μM TAPI‐0 inhibitor), and percentage of residual activity was calculated by comparison, considering 100% the activity of enzyme without inhibitor (n = 3). (*p  <  0.05, ***p ≤ 0.0001; one‐way ANOVA with Bonferroni Multiple Comparison Test, data are means ± SEM). Time course of human TIMP3 concentration after intravenous injection in DBA/2J mice of 2 mg/kg G3C12‐NTIMP3 peptide in (B) renal cortex and (C) serum (n = 3). (*p  <  0.05, **p ≤ 0.01, ***p ≤ 0.0001; one‐way ANOVA with Dunnett's Multiple Comparison Test referred to t 0, data are means ± SEM)

We created two new peptides specifically directed to the kidney, G3C12‐NTIMP3, and G3C12‐T2GNTIMP3, obtained by a conjugation of G3C12 carrier peptide with the N‐terminal TIMP3 domain, in a native form containing the inhibitor activity sites for MMPs and ADAM17 (NTIMP3), or in a form with a point mutation (T2G) described as able to uphold the inhibitory activity of ADAM17 (T2GNTIMP3),^23^ to evaluate if kidney‐specific delivery and accumulation of systematically administered human TIMP3 N‐terminal domain may slow progression of DN in mice. First, we performed specific enzymatic inhibitor screening assays to test the ability of G3C12, G3C12‐NTIMP3, and G3C12‐T2GNTIMP3 peptides to inhibit ADAM17 and Gelatinases MMP2 and MMP9 in vitro. The results showed that G3C12‐T2GNTIMP3 but not G3C12‐NTIMP3 was able to inhibit MMP2 (p < 0.05), whereas both NTIMP3 derived peptides showed similar significant inhibitory activity against MMP9 (p < 0.001) and ADAM17 enzyme (p < 0.001) (Figure 5A). As expected G3C12 peptide did not show any inhibitory activity, indicating that the inhibition is specifically due to the TIMP3 sequences. Peptide concentration has been chosen based on G3C12‐NTIMP3 dose dependently (0.5, 1 and 5 μM) inhibition of ADAM17 and MMP9, with the highest activity observed at 1μM (Figure S4A).

For pharmacokinetics in vivo, first DBA/2J mice, a high responder strain in STZ‐induced model of DN,^29^ were iv, ip, or sc injected with 2 mg/kg of G3C12‐NTIMP3 peptide or PBS as a control. After 24 hours, protein samples extracted from renal cortex showed higher peptide expression in iv injected mice (Figure S4B). Dose dependent peptide expression has been evaluated in renal cortex 24 hours after iv injection of 0.5, 1, or 2 mg/kg G3C12‐NTIMP3 peptide, and 2 mg/kg has been chosen for further experiments (Figure S4C). Twenty‐four hours after iv injection of 2 mg/kg G3C12‐NTIMP3, peptide expression in liver, pancreas, muscle, kidney medulla, and aorta was undetectable (Figure S4D). Next, renal cortex was collected at 0, 10′, 30′, 3, 6, 24, 48, and 72 hours after iv injection of 2 mg/kg G3C12‐NTIMP3 peptide, and the levels of NTIMP3 derived peptide was evaluated by an ELISA kit specific for detection of human TIMP3. Peptide expression was detectable in renal cortex starting from 30′ up to 48 hours after injection (Figure 5B). TIMP3 expression was evaluated also in serum, resulting significantly increased only 10′ after injection (Figure 5C). Similar experiments have been performed with (KKEEE)_3_K carrier conjugated with the T2GNTIMP3 peptide. (KKEEE)_3_K‐T2GNTIMP3 peptide showed significant inhibitory activity in vitro only for MMP9 enzyme (Figure S4E), and peptide expression was detectable in renal cortex 6 hours after injection, as assessed by ELISA assay (Figure S4F).

### 3.4 TIMP3 targeting delivery to the kidney reduces diabetic nephropathy in mice

**FIGURE 6:**
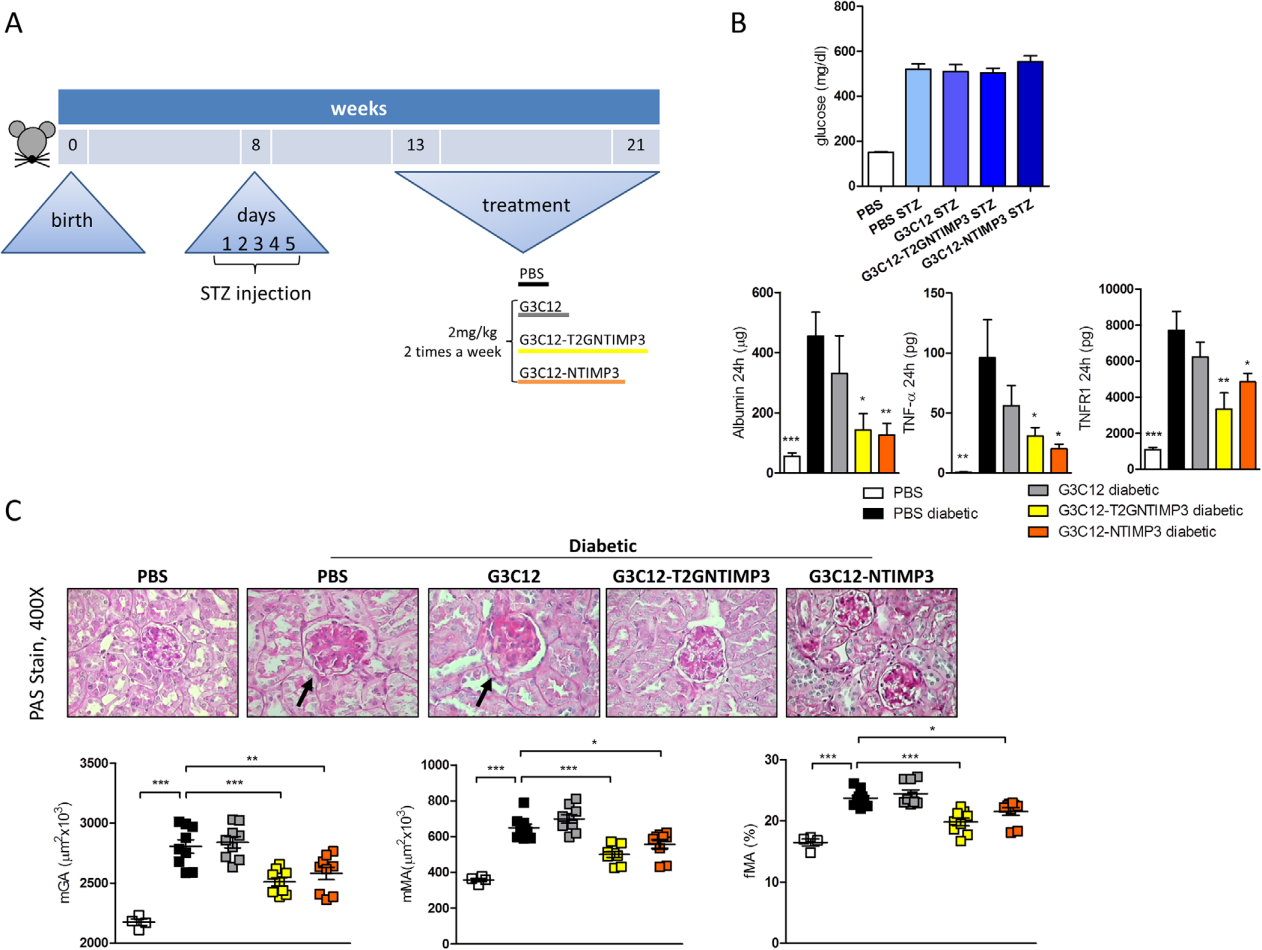
Human TIMP3 derived peptides treatment in diabetic mice. (A) DBA/2J mice were rendered diabetic at 8 weeks of age with a low‐dose STZ protocol. Four weeks after the day of diabetes onset, PBS, G3C12 free peptide, G3C12‐T2GNTIMP3 peptide, and G3C12‐NTIMP3 peptide were administered by iv injection for 8 weeks at a mean time of two injections per week at the concentration of 2 mg/kg. (B) Blood glucose levels, 24‐hour urinary albumin, TNF‐α, and TNFR1 levels and (C) PAS‐staining and quantification of mGA, mMA, and fMA in non‐diabetic DBA/2J mice (PBS) and in diabetic DBA/2J mice treated with PBS, G3C12 free peptide, G3C12‐T2GNTIMP3 peptide, or G3C12‐NTIMP3 peptide; (arrows indicate glomerular hypertrophy and mesangial expansion) (non‐diabetic n = 4, diabetic n = 9 per group, original magnification, X400). (*p  <  0.05, **p≤ 0.005 ***p ≤ 0.001; one‐way ANOVA with Dunnett's Multiple Comparison Test referred to PBS diabetic, data are means ± SEM)

**FIGURE 7:**
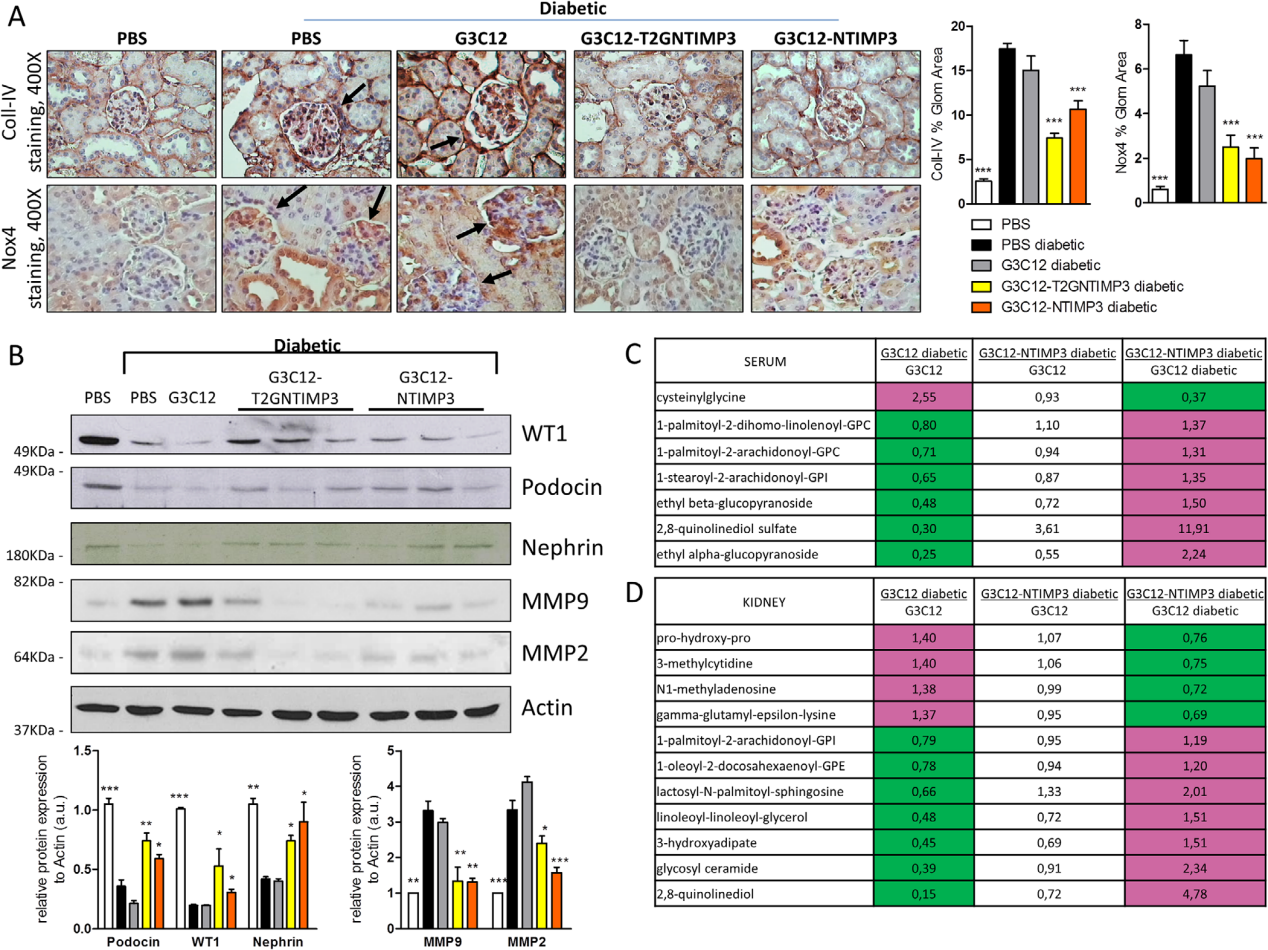
TIMP3 targeting delivery to the kidney reduces diabetic nephropathy in mice. (A) Immunohistochemical detection of Coll‐IV and Nox4 in kidney (original magnification, X400) (arrows indicate increased glomerular staining for Nox4 and Coll‐IV); and (B) protein expression of WT1, Podocin, Nephrin, MMP9, MMP2, and actin in kidney cortex from non‐diabetic DBA/2J mice (PBS) and diabetic DBA/2J mice treated with PBS, G3C12 free peptide, G3C12‐T2GNTIMP3 peptide, or G3C12‐NTIMP3 peptide. Relative protein levels as determined by densitometry. A representative image of respectively 1, 1, 1, 3, and 3 mice per group is shown. (non‐diabetic n = 4, diabetic n = 9 per group; *p  <  0.05, **p ≤ 0.005, ***p ≤ 0.001; one‐way ANOVA with Dunnett's Multiple Comparison Test referred to PBS diabetic, data are means ± SEM). Hierarchical clustering of metabolites significantly modulated in G3C12 but not in G3C12‐NTIMP3 peptide treated diabetic mice, compared to G3C12 non‐diabetic mice in (C) serum and (D) kidney cortex. Columns represent the different samples analyzed and rows represent the indicated metabolites. Metabolites expressed at lower levels are in green; those expressed at high levels are in magenta; fold changes are indicated in the right. Following log transformation and imputation with minimum observed values for each compound, Welch's two‐sample *t*‐test was used to identify biochemicals that differed significantly between experimental groups. (n = 6 per group). Abbreviation: a.u., arbitrary unit

To test the effect of peptides in a mouse model of Type 1 diabetes, DBA/2J mice were rendered diabetic at 8 weeks of age with a low‐dose STZ protocol. Four weeks after STZ injections, PBS, G3C12 peptide, G3C12‐NTIMP3 peptide, and G3C12‐T2GNTIMP3 peptide were administered by iv injection for 8 weeks at a mean time of two injections per week at the concentration of 2 mg/kg (Figure 6A). No toxic effects were observed in any of the treated groups. Neither have any changes been observed in the behavioral or nutritional status of the treated mice. Blood glucose levels increased following STZ and at the end of treatments were similar in all groups (Figure 6B). Prior to sacrifice, mice were placed into metabolic cages for a 24 hours urine collection. G3C12‐NTIMP3‐ and G3C12‐T2GNTIMP3‐treated mice showed significantly reduced albumin, TNF‐α, and TNFR1 levels in the urine compared to PBS or G3C12 treated mice (Figure 6B). At the end of the treatment, mice were euthanized and kidney analyzed by PAS staining for the quantification of renal lesions. mGA, mMA, and fMA resulted reduced in diabetic mice treated with G3C12‐NTIMP3 or G3C12‐T2GNTIMP3 compared to PBS and G3C12 treated mice (Figure 6C). Moreover, both G3C12‐NTIMP3 and G3C12‐T2GNTIMP3 treatments were associated with the reduction of ECM accumulation and glomerular damage, as determined by Coll‐IV and Nox4 staining (Figure 7A), and to the improvement of podocyte structural markers, such as WT1, Podocin and Nephrin and reduction of MMP9 and MMP2, as assessed by western blot (Figure 7B). Noteworthy, neither T2GNTIMP3 peptide without G3C12 carrier, nor T2GNTIMP3 peptide coniugated with (KKEEE)_3_K kidney carrier peptide ((KKEEE)_3_K‐T2GNTIMP3) provided renal protection in DBA/2J diabetic mice, as indicated by unchanged 24 hours urinary albumin, kidney morphology, or podocyte structural markers, compared to PBS injected diabetic mice (n = 9 mice per group) (Figures S5A‐S5D). The obtained results indicate that G3C12‐NTIMP3 and G3C12‐T2GNTIMP3 peptides may delay the progression of DN in mice, representing a valid therapeutic strategy. Next, we have performed untargeted metabolome profiling in sera and renal cortex extracts from diabetic mice treated with G3C12 (G3C12 diabetic), G3C12‐NTIMP3 peptide‐treated diabetic mice, and control non‐diabetic mice (G3C12), to screen for major molecular differences associated with G3C12‐NTIMP3 peptide treatment. Principle component analysis conducted on all groups in both serum and renal cortex showed that diabetic mice treated with G3C12 or G3C12‐NTIMP3 peptide samples were clustered together, while G3C12 non‐diabetic mice samples segregated clearly from the other groups. As expected, the diabetic condition resulted in the majority of differences at the global level.

While diabetes affected metabolism on several pathways compared with control, we observed that G3C12‐TIMP3 treatment was able to completely rescue diabetes‐associated changes in the levels of seven serum metabolites and 11 kidney metabolites, suggesting an association of these metabolites with deregulation of TIMP3 expression or activity. Interestingly, in kidney cortex, G3C12‐NTIMP3 treatment in diabetic mice counteracted the diabetes‐related induction of Prolyl‐hydroxy‐proline (pro‐hydroxy‐pro) and gamma‐glutamyl‐epsilon‐lysine, two markers of fibrosis and ECM deposition. In serum, the hydrolysis product of glutathione, cysteinylglycine disulfide, was strongly increased only in G3C12 diabetic mice, whereas G3C12‐NTIMP3 treatment during diabetes restored low levels similarly to non‐diabetic control (Figures 7C and 7D). Since gamma‐glutamyl‐epsilon‐lysine represents a crosslink product of transglutaminase type 2 (TG2) enzyme, next we evaluated TG2 expression and activity in kidney cortex. Results indicated that both were slightly increased in G3C12, but not in G3C12‐NTIMP3 diabetic mice, compared to non‐diabetic mice (Figure S6).

## 4 DISCUSSION

**FIGURE 8:**
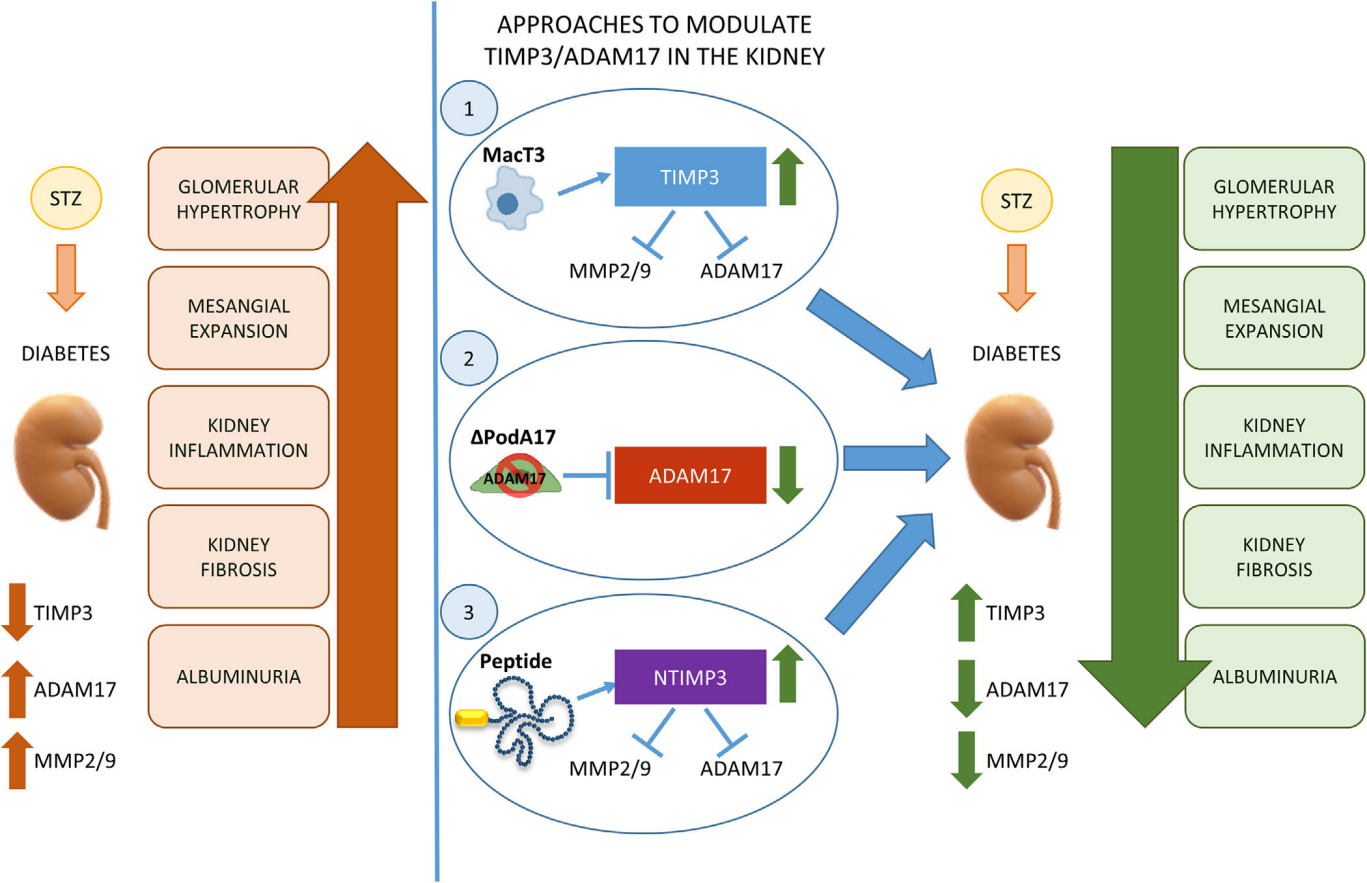
New approaches to modulate TIMP3/ADAM17 in the kidney. The loss of TIMP3 in animal models of diabetes leads to the exacerbation of diabetic renal injury in an organ‐specific manner. Human TIMP3 overexpression in myeloid lineage (1), conditional deletion of ADAM17 in podocytes (2), and intervention with innovative kidney‐targeted TIMP3 derived peptides (3), represent new strategies to modulate TIMP3 and its targets in kidney, resulting in protection from renal damage in experimental models of DN

In this work we provided evidences that in vivo manipulation of TIMP3/ADAM17 network specifically in diabetic kidney may limit the onset and the progression of DN. To this aim we analyzed diabetic mice (1) overexpressing human TIMP3 in myeloid lineage, (2) conditionally deleted for ADAM17 in podocytes, or (3) treated with new kidney targeted TIMP3 derived peptides. Obtained results showed that either genetic or pharmacological strategies may contribute to the reduction of albuminuria, glomerular hypertrophy, mesangial expansion, renal inflammation, and fibrosis induced by diabetes via regulation of MMP2, MMP9, and ADAM17 activities (Figure 8).

Studies in patients have implicated a possible role of TIMP3 in diabetic nephropathy.^30^, ^31^ The loss of TIMP3 in animal models of diabetes leads to the exacerbation of diabetic renal injury in an organ‐specific manner as demonstrated by mesangial expansion and increased albuminuria, associated with inflammation, fibrosis, and increased levels of Nox4, whose role in the development of renal damage and fibrosis associated with diabetes has been well demonstrated by multiple studies.^32^, ^33^, ^34^

Immunologic and inflammatory mechanisms are known to play a role in DN development and progression. We have previously generated a mouse model with overexpression of human TIMP3 under the control of CD68 promoter, (MacT3), to restrict TIMP3 expression to cells of myeloid lineage,^35^ and have already shown that MacT3 mice are protected from obesity‐induced inflammation and the progression of vascular damage associated with atherosclerosis. MacT3 macrophages showed increased levels of a functional TIMP3 protein when the promoter CD68 was activated and the ability to increase TIMP3 expression directly at the inflammatory sites.^21^, ^22^ Since renal monocyte infiltration is observed in the progressive course of both human and experimental DN,^36^ here we treated MacT3 mice with STZ and evaluated the effects in kidney disease induced by diabetes. In the current analysis, diabetic MacT3, and control mice showed a similar increase of infiltrating macrophages in kidney, compared to non‐diabetic mice. The preserved expression of TIMP3 in the renal cortex of diabetic MacT3 mice is explained by the overexpression of TIMP3 in transgenic macrophages, which compensated for the reduction of endogenous TIMP3 expression induced by the glucotoxic effect of diabetes, as observed in the kidney of diabetic WT mice. In fact, the overexpression of transgenic TIMP3 in MacT3 mice is under control of the CD68 promoter and unaffected by glucotoxicity.^21^, ^22^ Interestingly, preserved renal TIMP3 expression in diabetic MacT3 mice was associated with protection against diabetes‐induced kidney damage and albuminuria. A corollary observation of this study is that MacT3 mice were less susceptible to develop hyperglycemia after STZ treatment. This finding suggests that TIMP3 overexpression in cells of the myeloid lineage may confer an improved inflammatory profile in the endocrine pancreas, as multiple low doses STZ causes diabetes by destroying insulin‐producing β‐cells through inflammatory mechanisms.^37^ Future studies with appropriate models are needed to explain whether TIMP3 protects β‐cells from immune attack affecting apoptosis, regeneration, or other mechanisms. Overall, these results confirm the role for TIMP3 as a critical player in regulating the tissue microenvironment including control of inflammation and fibrosis,^5^, ^38^ as demonstrated by reduced inflammatory cytokines (Tnf‐α, IL6 and MCP1) and fibrotic markers (COL1A2, Coll‐IV and TGF‐β) in diabetic MacT3 mice. Production of mediators involved in these processes is known to be regulated by intracellular MAPK signalling.^39^ Our examination of MAPK activation in the diabetic kidneys found that TIMP3 overexpression caused significant attenuation of p38 phosphorylation. Clinical studies have demonstrated that kidney p38 MAPK activity is increased and associated with the development of DN.^40^ In diabetic animal models, p38 MAPK activity rapidly increases in glomeruli and tubules after the induction of hyperglycaemia, as well as in the accumulating kidney interstitial cells associated with advanced nephropathy.^41^ Studies of non‐diabetic kidney disease have shown that pharmacological inhibition of p38 MAPK suppressed inflammation and fibrosis.^42^, ^43^ TIMP3 overexpression in the kidney of MacT3 diabetic mice was also associated with conserved podocyte‐specific markers, suggesting a protective role of TIMP3 in diabetic podocytopathy, a critical player in the pathogenesis of glomerulosclerosis and proteinuria.^44^

To gain insight into the role of the local ADAM17, a direct target of TIMP3, in renal pathophysiology, we generated a new mouse model through conditionally inactivation of ADAM17 in podocytes ∆PodA17. We and others have shown that selective inhibition of ADAM17 in specific cell types could be beneficial for treatment of several pathologies.^26^, ^45^, ^46^ Our findings indicate that conditional deletion of ADAM17 in podocytes improves albuminuria and ameliorates progression of DN, protecting podocytes. Podocyte markers podocin, nephrin, and WT1, key factors for renal development and podocyte functions maintenance, were substantially ameliorated in ΔPodA17 diabetic mice compared to control diabetic mice, suggesting preserved podocyte architecture.^47^, ^48^, ^49^

Interestingly in ∆PodA17, we also found a significant reduction of both serum and urinary concentration of TNF‐α, accompanied by a reduction of albuminuria. TNF‐α synthesis and release are critical factors underlying the development of early pathological alterations during DN, including renal hypertrophy. Clinical studies have shown that serum and urinary TNF‐α levels are elevated in diabetic patients with increased urinary albumin excretion, urinary TNF‐α excretion increases as DN progresses and is associated with clinical markers of glomerular and tubulointerstitial damage. Moreover, administration of anti‐TNF‐α agents in diabetic rats significantly decreased urinary TNF‐α concentrations and prevented renal hypertrophy.^50^ Although different resident renal cells have been shown able to synthesize and release TNF‐α, the main source and stimuli of renal TNF‐α production remain to be definitively elucidated. Here, the evidences that we have found suggest that among glomerular cells, podocytes may be involved in this process. Overall, podocyte‐specific ADAM17 deletion improved diabetic glomerulopathy as demonstrated by reduced glomerular hypertrophy, mesangial expansion, and accumulation of Coll‐IV and Nox4 expression.

Recent evidence in pre‐clinical studies suggests that gene‐based therapy using TIMPs may have broad clinical potential, and a number of strategies have been tested for the development of TIMP3 delivery to inflammatory sites or in a cardiovascular setting.^51^, ^52^, ^53^ Here, we provide evidence that treatment with exogenous peptides, derived from the fusion of the N‐terminal domain of human TIMP3 protein with G3C12 galectin‐3 targeting peptide, may delay development of DN in DBA/2J diabetic mice. We focused the study on NTIMP3 domain because previous papers demonstrated that this portion of TIMP3 is convenient for protein‐engineering to increase MMPs and ADAM17 selectivity.^54^, ^55^ G3C12 has been proved to specifically accumulate in the kidneys after injection and has been already used successfully in conjugation with the angiotensin converting enzyme inhibitor, captopril.^19^ We compared the NTIMP3 form with a form with a point mutation (T2G) described as able to uphold the inhibitory activity of ADAM17 (T2GNTIMP3),^23^ however, once conjugated with G3C12 carrier, the two peptides showed in vitro similar inhibitory activity against both, ADAM17 and MMP9 whereas inhibition of MMP2 resulted compromised in NTIMP3 peptide.

In the present study, the treatment with peptides delayed development of nephropathy in in vivo model of diabetes, resulting in reduction of albuminuria, urinary TNF‐α and TNFR1 levels, membrane thickness, and mesangial expansion compared to diabetic control mice. Consistently, protein expression of podocytes markers is significantly improved in kidney cortex whereas fibrosis markers are reduced, reflecting the protective effects of TIMP3 on renal lesions, as observed in MacT3 and ∆PodA17 mice. Plasma glucose levels were not affected by peptides, indicating that the renoprotection was exerted through a mechanism independent from glycemic control.

Treatments of diabetic mice with free T2GNTIMP3 peptide or with T2GNTIMP3 conjugated with the (KKEEE)_3_K carrier, failed to succeed in terms of kidney injury improvement, suggesting the prominent role of G3C12 sequence as specific kidney carrier during diabetes.

Metabolomics relevance in discovering of predictive, diagnostic, and prognostic biomarkers of DN is increasing.^56^ Due to these advantages, we evaluated, by global metabolomics analysis, the changes of metabolic profiles in sera and kidney extracts from G3C12 or G3C12‐NTIMP3 peptide‐treated diabetic mice and G3C12 non‐diabetic mice, to identify TIMP3‐associated changes in plasma and kidney metabolites. As expected, diabetes resulted in the majority of differences at the global level, in particular in serum samples. However, seven serum metabolites and 11 kidney cortex metabolites were modulated only in G3C12 but not in G3C12‐NTIMP3 treated diabetic mice compared to non‐diabetic mice, suggesting an association with TIMP3 expression or activity deregulation. In serum, the increase in G3C12 diabetic mice of circulating levels of cysteinylglycine disulfide, a highly reactive metabolite which has been suggested to be involved in the production of reactive oxygen species and stimulation of oxidative reactions, may reflect the alterations of redox state of the system, being disturbances in redox status of plasma thiols associated with chronic renal failure. Moreover, total concentration of cysteinyl‐glycine was increased in predialysis CKD patient, and a generalized derangement in thiol redox status is considered a characteristic feature of uremia.^57^, ^58^ Metabolomics analysis in kidney samples show that pro‐hydroxy‐pro and gamma‐glutamyl‐epsilon‐lysine resulted increased only in G3C12 but not in G3C12‐NTIMP3 treated diabetic mice, compared to non‐diabetic control mice, supporting a protective role for G3C12‐NTIMP3 peptide in DN by restraining cellular fibrogenic responses. The increase of TG2 enzyme and its crosslink product gamma‐glutamyl‐epsilon‐lysine have been observed in diabetic kidney biopsies in humans and mice, associated with progressive kidney fibrosis.^59^ Moreover, TG2 inhibition ameliorates the progression of experimental diabetic nephropathy and can be considered for clinical application.^60^, ^61^ Here, we show a significant reduction of TG2 activity associated with G3C12‐NTIMP3 peptide treatment during diabetes; however, the link between TIMP3 and TG2 should be more investigated to evaluate if TIMP3 may control, directly or through the regulation of its targets, the activity of TG2, thus modulating kidney fibrosis. Pro‐hydroxy‐pro represents a collagen breakdown product, and we can speculate that its reduction in G3C12NTIMP3‐treated mice may be related to the reduced activity of gelatinases MMP2 and MMP9 observed in kidney cortex. 2,8‐quinolinediol, a microbiota‐derived hydroxylated quinoline derivative, was the unique metabolite with reduced levels in both serum and kidney of G3C12 diabetic mice, compared to non‐diabetic and G3C12‐NTIMP3 treated diabetic mice. However, the mechanism regarding how G3C12‐NTIMP3 treatment can cause a decrease in 2,8‐dihydroxyqunoline warrants further investigation.

## 5 CONCLUSIONS

Despite the progress due to intensive lifestyle approach combined with careful metabolic control and availability of new drugs such as glifozins, diabetic nephropathy is still missing a treatment aiming at correcting its biology. Our data demonstrate that in in vivo model of long‐term diabetic renal pathology, restoring high TIMP3 activity specifically in the kidney, may represent a therapeutic strategy for the amelioration of renal damage under conditions, including diabetic nephropathy, in which its reduction is related to the disease onset and progression.

TIMP3 effects are exerted through a mechanism independent from glycemic control and may contribute to a significant improvement in the effectiveness of the treatments to prevent and/or reduce the progression of diabetic nephropathy, one of the top three causes of terminal kidney disease in the Western world. Interestingly, we also provided evidence that circulating metabolites are potential companion biomarkers of the therapeutic efficacy. The new therapeutic approach together with companion biomarkers has a clear clinical implication.

Furthermore, given the prevalence of DN in patients undergoing dialysis, our therapeutic approach might translate into a significant health and economic impact.

In conclusion, TIMP3 fusion peptide injections may represent an alternative, less invasive approach, with high eligibility and accessibility and significantly cost saving.

## CONFLICT OF INTEREST

Viviana Casagrande, Stefano Menini, Massimo Federici, and Rossella Menghini are authors of a patent issued for the Timp3 peptides and the targeting approach related to this work. The rest of authors declare no conflict of interest.

## AUTHOR CONTRIBUTIONS

Conceptualization: Massimo Federici and Rossella Menghini. Experimental investigation: Viviana Casagrande, Giulia Iuliani, Stefano Menini, and Rossella Menghini. Data Analysis, Viviana Casagrande, Stefano Menini, and Rossella Menghini. Writing – original draft: Rossella Menghini. Writing – review and editing: Rossella Menghini, Viviana Casagrande, Stefano Menini, Giuseppe Pugliese, and Massimo Federici. Supervision: Massimo Federici. Project Administration: Massimo Federici. Funding acquisition: Massimo Federici and Rossella Menghini. All authors revised the article and approved the final version to be published. All authors had full access to all the data in the study and accept responsibility to submit for publication.

## Supporting information

Supporting information.

## Data Availability

The data used in the present study are available from the corresponding author on reasonable request.
