# Supplementary material for: Restoration of renal TIMP3 levels via genetics and pharmacological approach prevents experimental diabetic nephropathy
Source: Clin Transl Med. 2021 Feb 4;11(2):e305. doi: 10.1002/ctm2.305 (PMC7862169; doi:10.1002/ctm2.305)
Supplement: Supplementary file 1 — Supporting information. [file CTM2-11-e305-s001.docx]

**Restoration of renal TIMP3 levels via genetics and pharmacological approach prevents experimental diabetic nephropathy**

Viviana Casagrande^1,2^, Giulia Iuliani^1^, Stefano Menini^3^, Giuseppe Pugliese^3^, Massimo Federici^1,4^*, Rossella Menghini^1^*

^1^ Departments of Systems Medicine, University of Rome “Tor Vergata,” Rome, Italy.

^2^ Research Unit of Diabetes and Endocrine Diseases, Fondazione IRCCS “Casa Sollievo della Sofferenza”, San Giovanni Rotondo, Italy.

^3^ Department of Clinical and Molecular Medicine, “Sapienza” University, Rome, Italy.

^4^ Center for Atherosclerosis, Department of Medical Sciences Policlinico Tor Vergata University, Rome, Italy.

*Corresponding authors: E-mail: [menghini@med.uniroma2.it](mailto:menghini@med.uniroma2.it) (R.M.); [federicm@uniroma2.it](mailto:federicm@uniroma2.it) (M.F.)

**Supporting information**

Figure S1. Diabetes-induced CD68 positive cells infiltration in renal cortex of wt and MacT3 mice.

Figure S2. Negative and isotype controls for NOX4 staining

Figure S3. Diabetes-induced glomerular apoptosis in ∆PodA17 mice

Figure S4. Pharmacokinetics analysis of G3C12-NTIMP3 and (KKEEE)_3_K-T2GNTIMP3 peptides.

Figure S5. Effect of T2GNTIMP3 and (KKEEE)_3_K-T2GNTIMP3 treatments in diabetic mice.

Figure S6. Gene expression and activity of Transglutaminase 2 in Kidney cortex

**Figure S1. Diabetes-induced CD68 positive cells infiltration in renal cortex of wt and MacT3 mice.**

Immunohistochemical detection of CD68 in kidney of wt and MacT3 non diabetic and diabetic mice (n=5 per group), 20 random fields of the renal cortex were examined at a final magnification of 400X and the results were expressed as the mean number of CD68 positive cells per field.

**Figure S2. Negative and isotype controls for NOX4 staining.**

Imunohistochemical detection of Nox4 in **A)** normal liver tissue (negative control), and in **B)** diabetic kidney (positive control); **C)** immunohistochemical analysis in diabetic kidney with the recombinant Rabbit IgG (Isotype Control). Original magnification, X100 or X400, as indicated in the figure.

**Figure S3. Diabetes-induced glomerular apoptosis in ∆PodA17 mice.**

Immunohistochemical detection of Active caspase-3 in kidney of Ct and ΔPodA17 non diabetic and diabetic mice (n=5 per group). (*p < 0.05; Student’s t test comparing diabetic mice, data are means ± SEM). Positive cells for active-caspase-3 were counted and expressed as percent of total glomerular cells (labeling index).

**Figure S4. Pharmacokinetics analysis of G3C12-NTIMP3 and (KKEEE)_3_K-T2GNTIMP3 peptides.**

**A)** G3C12-NTIMP3 dose dependently (0.5, 1 and 5 µM) inhibition of ADAM17 and MMP9 activity in vitro (n=3; ***p ≤ 0.001; one-way ANOVA with Dunnett’s Multiple Comparison Test referred to PBS, data are means ± SEM). **B)** Protein expression of G3C12-NTIMP3 and Actin in kidney cortex from non-diabetic DBA/2J mice after 24 h from intravenously (iv), intraperitoneally (ip), or subcutaneously (sc) injection of 2mg/kg of G3C12-NTIMP3 peptide or PBS (iv) as a control and **C)** protein expression of G3C12-NTIMP3 and Actin in in kidney cortex from non-diabetic DBA/2J mice after 24 h from iv injection of 0.5, 1 or 2mg/kg of G3C12-NTIMP3 peptide or PBS as a control (n=3 mice per group). A representative image of 2 mice per group is shown. **D)** Protein expression of G3C12-NTIMP3 and Actin in kidney cortex, liver, pancreas, muscle, kidney medulla, and aorta, from non-diabetic DBA/2J mice after 24 h from intravenously injection of 2mg/kg G3C12-NTIMP3 peptide (n=3 mice per group; a representative image of 1 mice per group is shown). **E)** Inhibition of human MMP9, MMP2 and ADAM17 enzyme activity by (KKEEE)_3_K and (KKEEE)_3_K-T2GNTIMP3 in vitro. Enzymes were incubated or not with the various peptides (1µM) or positive control (1,3µM NNGH inhibitor or 1µM TAPI-0 inhibitor) and percentage of residual activity was calculated by comparison, considering 100% the activity of enzyme without inhibitor (n=3). **F)** Renal cortex time course of human TIMP3 concentration after intravenous injection of 2mg/kg (KKEEE)_3_K-T2GNTIMP3 peptide in mice (n= 3). (*p < 0.05, **p ≤ 0.01, ***p ≤ 0.001; one-way ANOVA with Bonferroni Multiple Comparison Test, data are means ± SEM).

**Figure S5. Effect of T2GNTIMP3 and (KKEEE)_3_K-T2GNTIMP3 treatments in diabetic mice.**

DBA/2J mice, were rendered diabetic at 8 weeks of age with a low-dose STZ protocol. 4 weeks after the day of diabetes onset, PBS, T2GNTIMP3 or (KKEEE)_3_K peptide were administered by iv injection and (KKEEE)_3_K-T2GNTIMP3 were administered by iv or ip injection, as indicated, for 8 weeks at a mean time of two injections per week at the concentration of 2mg/kg. **A)** Quantification of mGA, fMA and mMA evaluated by PAS-staining and **B)** kidney cortex protein expression of Podocin, WT1, and Actin in non-diabetic DBA/2J mice (PBS) and in diabetic DBA/2J mice treated with PBS, or T2GNTIMP3 peptide. (non diabetic n=4, diabetic n=9 per group). **C)** 24-h urinary albumin and **D)** kidney cortex protein expression of Podocin, WT1, and Actin in non-diabetic DBA/2J mice (PBS) and in diabetic DBA/2J mice treated with PBS, (KKEEE)_3_K, (KKEEE)_3_K-T2GNTIMP3 (iv or ip) peptide. (non diabetic n=4, diabetic n=9 per group).

**Figure S6. Gene expression and activity of Transglutaminase 2 in kidney cortex.**

**A)** Gene expression of Transglutaminase 2 level and **B)** Transglutaminase 2 activity in kidney cortex analysis from non-diabetic DBA/2J mice treated with G3C12 free peptide and diabetic DBA/2J mice treated with G3C12 free peptide or G3C12-NTIMP3 peptide. (n=9 per group) (*p < 0.05, **p≤ 0.005 ***p ≤ 0.001; one-way ANOVA with Bonferroni Multiple Comparison Test, data are means ± SEM). a.u., arbitrary unit. TG2 activity was evaluated in 10 µg of kidney cortex extracts using specific assay kit, according to the manufactures instruction (Novus Biologicals).
